# Marginal secondary displacement in fractures of the distal radius at follow-up – an important predictor for late displacement and malunion

**DOI:** 10.1177/17531934221146063

**Published:** 2023-01-09

**Authors:** Viktor Schmidt, Cecilia Mellstrand-Navarro, Sebastian Mukka, Mats Wadsten

**Affiliations:** 1 Department of Surgical and Perioperative Sciences at Umeå University, Sweden; 2 Department of Clinical Science and Education, Södersjukhuset, Karolinska Institutet, Stockholm, Sweden; 3 Department of Hand Surgery, Södersjukhuset, Stockholm, Sweden

**Keywords:** Distal radius fracture, marginal secondary displacement, instability, malunion, late displacement

## Abstract

Treatment recommendations in fractures of the distal radius are often based on the degree of displacement and functional demands. The fracture may be within an acceptable radiological range, but a marginal deterioration in alignment then occurs between the initial visit and follow-up. This may pose a risk for late displacement that may require further treatment. We secondarily analysed prospectively collected data and included 165 patients. We found that marginal secondary displacement (odds ratio (OR) 9.7), anterior comminution (OR 8.8), loss of anterior apposition (OR 6.8) and dorsal comminution (OR 2.6) were predictors of late displacement. Marginal secondary displacement is an important predictor of late displacement and malunion in fractures of the distal radius. Clinicians should not unequivocally accept general guidelines on alignment but also assess a deterioration in fracture alignment on radiographic follow-up and be aware of the potential need for surgery to avoid malunion in cases that show early secondary displacement, even when radiographic measures are within acceptable limits.

**Level of evidence:** III

## Introduction

Treatment of distal radial fractures (DRFs) largely depends on radiographic fragment displacement and on the functional demands of the patient (Johnson and Dias, 2019; Kvernmo, 2015; Lichtman et al., 2011; Mellstrand Navarro, 2021; Nederlandse Vereniging voor Heelkunde, 2010; Schmidt et al., 2022; Sundhedsstyrelsen, 2014). Fractures with minimal displacement after reduction (defined as <10° of dorsal tilt, >15° of radial inclination and <2 mm of ulnar variance) (Johnson and Dias, 2019; Kvernmo, 2015; Lichtman et al., 2011; Mellstrand Navarro, 2021; Nederlandse Vereniging voor Heelkunde, 2010; Schmidt et al., 2022; Sundhedsstyrelsen, 2014) are generally recommended for initial non-operative treatment with cast and subsequent outpatient radiographic follow-up (Arora et al., 2011; Earnshaw et al., 2002; Kumar et al., 2008; Mackenney et al., 2006). Potentially unstable DRFs are challenging to identify, and malunion is a well-known complication (Mackenney et al., 2006; Sirniö et al., 2019; Wadsten et al., 2014). Several predictors of displacement have been suggested, including female sex, advanced age, loss of anterior apposition and anterior and dorsal comminution (Walenkamp et al., 2016).

The decision to recommend surgical treatment at outpatient follow-ups is often based on the degree of radiological displacement and the functional demands of each patient (Johnson and Dias, 2019; Kvernmo, 2015; Lichtman et al., 2011; Mellstrand Navarro, 2021; Nederlandse Vereniging voor Heelkunde, 2010; Schmidt et al., 2022; Sundhedsstyrelsen, 2014). The deterioration of fracture position since the initial radiographs may predict late displacement and malunion. If the fracture is within the acceptable range but has marginal secondary displacement between the initial visit and the follow-up, there could be a risk of further deterioration resulting in late, substantial displacement (Mellstrand Navarro, 2021; Schmidt et al., 2022).

This study investigates whether DRFs' marginal secondary displacement is a predictor of late displacement. Secondarily, we sought to determine risk factors for late displacement.

## Methods

This study was a retrospective analysis of prospectively collected, multicentre data (Wadsten et al., 2014) that included patients treated for a DRF between October 2009 and September 2011 at two secondary-level hospitals in Sweden with catchment areas of approximately 160,000 and 130,000 inhabitants, respectively. Strengthening the Reporting of Observational Studies in Epidemiology guidelines were strictly followed.

### Participants and data collection

Patients aged 15–75 years with closed physes presenting with an acute DRF were screened for participation. Data were collected prospectively by follow-up visits (10–14 days, 3 months and 1 year after injury) at the participating orthopaedic departments. Patient data included age, sex, dominant side and initial and definitive treatment (non-operative/operative) (Table 1). Exclusion criteria were previous fracture of the same wrist, coexisting carpal fracture or scapholunate advanced collapse of the wrist, rheumatoid arthritis, alcohol or drug abuse, open fracture, dementia, unfused distal radial epiphysis, neurological impairment and Galeazzi's fracture.

**Figure 1.**
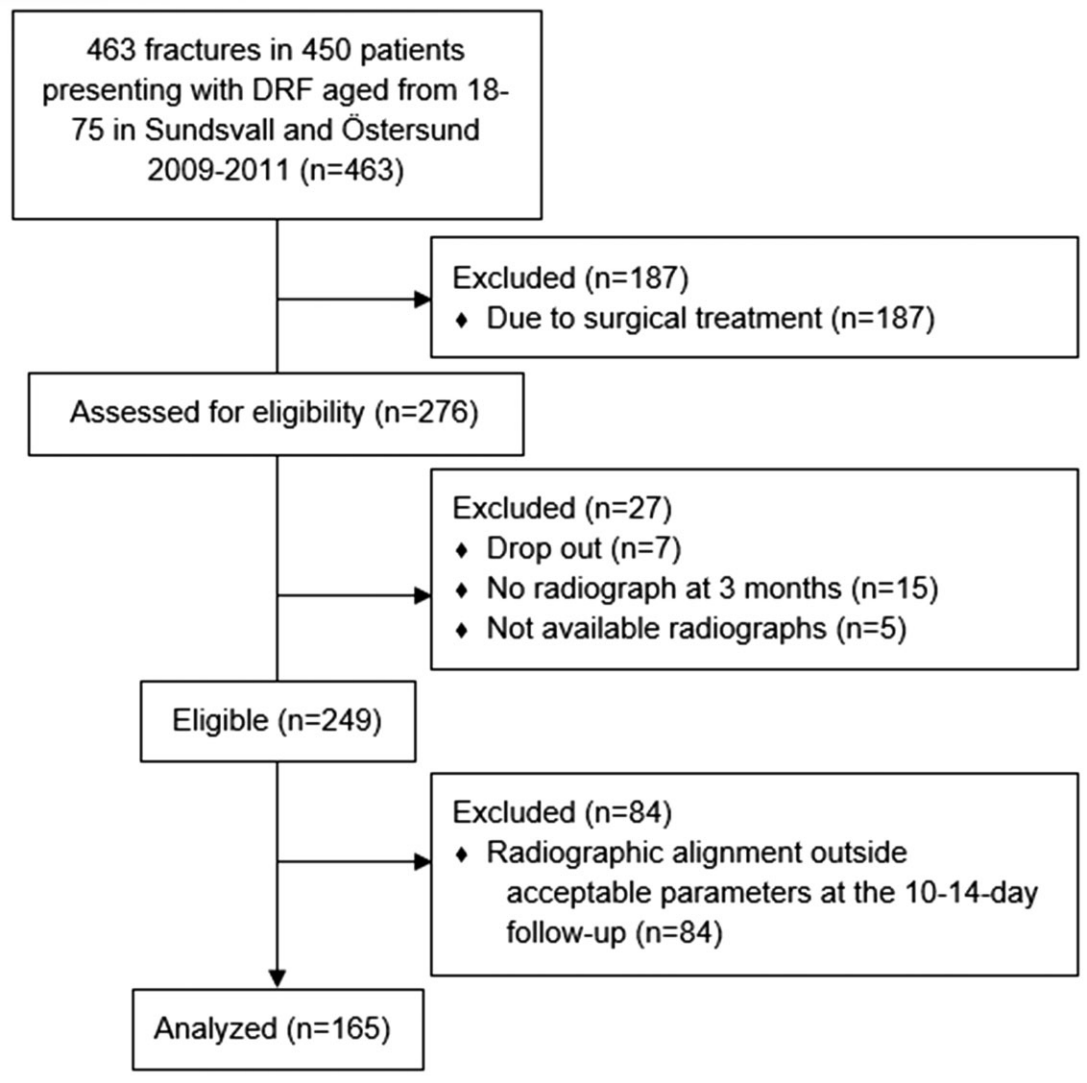
Flowchart of patients' inclusion and exclusion in a secondary analysis of prospectively collected data of 165 DRFs between October 2009 and September 2011 at the Departments of Orthopedic Surgery in *x* and *y*, Sweden.

For the present analysis, we selected fractures treated non-operatively (with dorsal, below elbow cast for 5 weeks, followed by rehabilitation with an occupational therapist) that had a complete radiological follow-up. Patients with radiographic alignment outside acceptable parameters defined by our protocol at 10–14 days after injury were excluded (Figure 1).

**Table 1.** Background data on 165 non-operatively treated patients with a distal radial fracture between October 2009 and September 2011 at the Departments of Orthopedic Surgery in Sundsvall and Östersund, Sweden.

Number of patients (% female)	165 (70%)
Age	57 (IQR 45–64; range 17–75)
Dominant hand	
Right	143 (87%)
Left	8 (5%)
NA*	14 (8%)
Hospital	
Sundsvall	75 (45%)
Östersund	90 (55%)
Reduced	63 (38%)
Dorsal comminution	71 (43%)
Anterior comminution	8 (5%)
Loss of anterior apposition	13 (8%)
Marginal secondary displacement	28 (17%)
Late displacement	35 (21%)

* NA = missing data concerning dominant hand.

### Radiographic measurements

**Figure 2.**
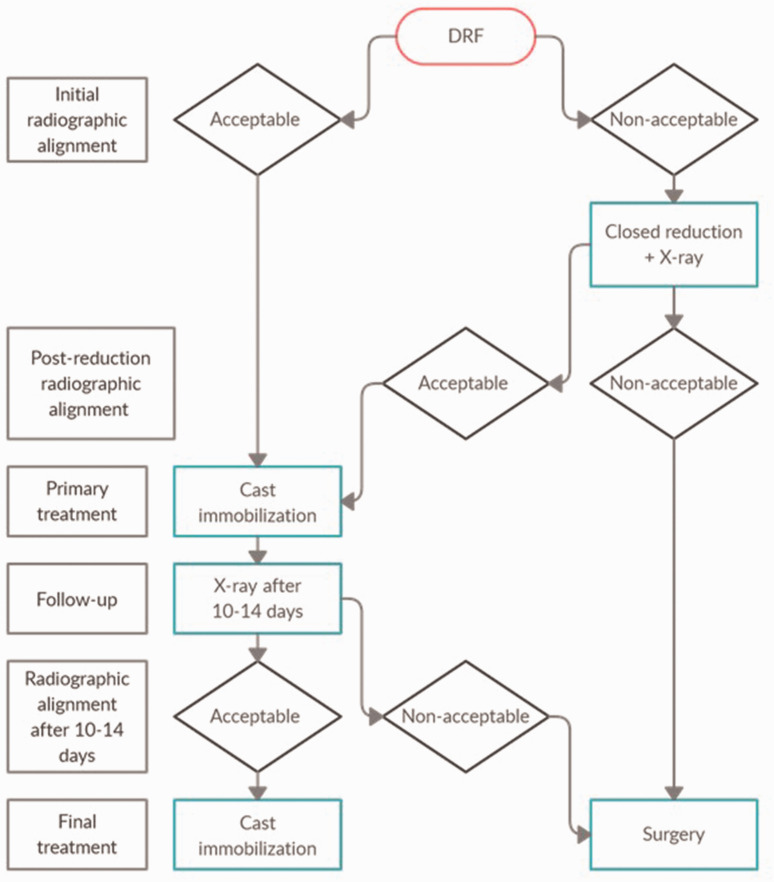
Treatment algorithm used in a secondary analysis of prospectively collected data of 165 DRFs between October 2009 and September 2011 at the Departments of Orthopedic Surgery in *x* and *y*, Sweden.

Radial inclination, anterior tilt, ulnar variance and intra-articular step-off were measured on anteroposterior and lateral radiographs (wrist in 15° elevation) in neutral rotation. Images were performed at the initial visit before and after reduction, 10–14 days after injury, and after union at a minimum of 3 months. There were no nonunions. Dorsal and anterior comminution were assessed in the lateral view and defined as a free-floating piece of the cortex. Small fragments of ≤3 mm were not considered comminution (Wadsten et al., 2017). We used a treatment algorithm (Figure 2) similar to Abramo et al. (2008). Acceptable alignment for non-operative treatment was defined as anterior tilt <20°, dorsal tilt <10°, radial tilt >10° and positive ulnar variance <2 mm. This secondary follow-up complements the data with precise measurements of radiographic alignment (measured from the central reference point (Medoff, 2005)) and measurements of anterior apposition (LaMartina et al., 2015; Phillips and Al-Shawi, 2014) (alignment of the anterior cortex, which we defined as *ad latus* displacement in the anteroposterior direction of no more than one cortical width) to allow for further analysis. All images were digitally acquired using the Picture Archiving and Communication System (Impax: Agfa, Mortsel, Belgium). All measurements were made by the first author.

### Outcomes

We analysed whether marginal secondary displacement on radiographs between the emergency visit and 10–14 days after injury was a risk factor for late displacement.

We defined marginal secondary displacement as acceptable alignment (as described above) despite a deterioration (i.e. a change to the worse between the emergency visit and at 10–14 days post-injury) of ≥5° in dorsal tilt, ≥2 mm of ulnar variance or ≥5° of loss in radial inclination.

Secondary displacement was defined as displacement (outside acceptable alignment) between the emergency visit and 10–14 days after injury. Late displacement was defined as displacement between 10–14 days after injury and final union at 3 months (Appendix 1).

### Statistics

Numerical data were tested for normality with the Shapiro–Wilks test. Skewed numerical data were presented as medians with interquartile range and proportions as percentages. A logistic regression model was used to assess the risk of late displacement. Assumptions for logistic regression were met. The variables sex, age, anterior apposition, anterior comminution, dorsal comminution and marginal secondary displacement were included in the model (Table 2). All variables were dichotomized. Age was divided into two groups: ≤55 and >55 years.

We examined late deterioration and split it into three components (dorsal tilt, radial inclination and ulnar variance). Deterioration was dichotomized for both early (at 10–14 days) and late (3 months) deterioration to ≥5° concerning dorsal tilt and radial inclination and to ≥2 mm for ulnar variance. We used sex, age, anterior apposition, anterior comminution, dorsal comminution, early deterioration in dorsal tilt, early deterioration in radial inclination and early deterioration in ulnar variance in our three models (Table 3). Results were presented as odds ratios (ORs) and their 95% confidence interval. No power calculation was made before this study. A retrospective ad-hoc power calculation found that 13 patients in each group would be enough to detect a difference regarding marginal secondary displacement. A two-sided *p*-value of <0.05 was considered statistically significant.

The study was conducted was approved by the regional ethical committee at Umeå University (Dnr 09-213). Written informed consent was obtained from all patients.

## Results

We enrolled 463 fractures in 450 patients. Two hundred seventy-six fractures were treated non-operatively. Of these, 249 (54%) had a complete follow-up with radiographs and were included. In five cases, the radiographs at the 10–14-day follow-up were no longer available in our archives and thus could not be re-evaluated. We lost 15 patients for follow-up who did not have a radiograph after 3 months. Seven patients opted to drop out of the study.

Patients (*n* = 84) with radiographic alignment outside acceptable parameters 10–14 days after injury were excluded. Thus, 165 patients were treated non-operatively with complete follow-up and acceptable alignment at the 10–14-day follow-up as defined by our protocol (Figure 1). There were 116 (70%) women and 49 (30%) men, with a median age of 57 years (range 18–75) (Table 1).

### Outcomes

Dorsal comminution, anterior comminution, loss of anterior apposition and marginal secondary displacement were significant predictors of late displacement. Age (over/under 55 years) and sex were not significant predictors. The parameter with the highest OR for predicting displacement was marginal secondary displacement, followed by anterior comminution and loss of anterior apposition (Table 2).

**Table 2.** Multivariate analysis with logistic regression for late displacement in 165 non-operatively treated patients with distal radial fractures in 2009–2011 in Sundsvall and Östersund, Sweden.

	*N*	Minimally displaced^a^	Late displacement^b^	Crude		Adjusted	
				OR^c^	2.5% to 97.5%	OR	2.5% to 97.5%
Sex							
Female	116	82 (71%)	34 (29%)	ref	–	ref	–
Male	49	38 (78%)	11 (22%)	0.7	0.3 to 1.5	0.9	0.3 to 2.4
Age >55							
No	74	59 (80%)	15 (20%)	ref	–	ref	–
Yes	91	61 (67%)	30 (33%)	1.9	1.0 to 4.0	1.5	0.6 to 3.8
Dorsal comminution							
No	94	78 (83%)	16 (17%)	ref	–	ref	–
Yes	71	42 (59%)	29 (41%)	3.4	1.7 to 7.0	2.6	1.1 to 6.4
Anterior comminution							
No	157	118 (75%)	39 (25%)	ref	–	ref	–
Yes	8	2 (25%)	6 (75%)	9.1	2.0 to 63.7	8.8	1.5 to 73.4
Anterior apposition							
Yes	152	117 (77%)	35 (23%)	ref	–	ref	–
No	13	3 (23%)	10 (77%)	11.1	3.2 to 51.8	6.8	1.6 to 36.4
Marginal secondary displacement							
No	137	112 (82%)	25 (18%)	ref	–	ref	–
Yes	28	8 (29%)	20 (71%)	11.1	4.5 to 29.7	9.7	3.6 to 28.5

a Minimally displaced = healed with acceptable alignment.

b Late displacement = healed in malunion because of late displacement.

c OR = odds ratio.

We also found that marginal secondary displacement in dorsal tilt and dorsal comminution were statistically significant risk factors for late deterioration of dorsal tilt. Dorsal comminution and loss of anterior apposition were significant risk factors for late deterioration in radial tilt. The loss of the anterior apposition significantly affected late deterioration in ulnar variance (Table 3).

**Table 3.** Multivariate analysis with logistic regression for late radiological deterioration in 165 non-operatively treated patients with distal radial fractures in 2009–2011 in Sundsvall and Östersund, Sweden.

	Late deterioration dorsal tilt		Late deterioration radial inclination		Late deterioration ulnar variance	
	OR	2.5% to 97.5%	OR	2.5% to 97.5%	OR	2.5% to 97.5%
Sex	0.8	0.4 to 1.9	0.9	0.2 to 4.0	1.8	0.5 to 6.2
Age*	1.7	0.8 to 3.6	1.6	0.4 to 6.8	1.5	0.5 to 5.7
Dorsal comminution	2.2	1.1 to 4.6	9.0	1.9 to 71.3	2.5	0.7 to 9.4
Anterior comminution	1.0	0.1 to 5.9	1.8	0.1 to 16.8	2.9	0.3 to 17.8
Anterior apposition	0.3	0.0 to 1.3	12.4	2.6 to 65.6	7.8	1.8 to 33.6
Marginal secondary displacement						
Dorsal tilt	4.3	1.6 to 12.8	0.5	0.1 to 2.9	1.4	0.2 to 6.2
Radial inclination	–	–	–	–	–	–
Ulnar variance	1.0	0.0 to 13.5	1.4	0.0 to 22.1	2.8	0.1 to 42.4

Deterioration was dichotomized for both early and late deterioration to ≥5 or <5° in dorsal tilt and radial inclination and to ≥2 or <2 mm for ulnar variance

* Age was dichotomized at >55 or ≤55 years.

The intraclass correlation coefficient was calculated regarding intra-rater reliability on 25 cases for dorsal tilt (0.97, excellent), radial inclination (0.94, excellent) and ulnar variance (0.89, good) with 1 year between measurements.

## Discussion

This study identified marginal secondary displacement as an important predictor of late displacement in non-operatively treated DRFs. Anterior comminution, dorsal comminution and loss of anterior apposition were risk factors for late displacement and subsequent malunion.

Late displacement in DRFs occurs in as many as 31–33% of cases (Mackenney et al., 2006; Sirniö et al., 2019; Wadsten et al., 2014). Previous studies have shown that reduced DRFs deteriorate over time (Abbaszadegan et al., 1988; Altissimi et al., 1994). The degree of displacement can be used as a predictor for clinical outcome, where greater displacement is correlated with worse outcome.

Predictors of instability in DRFs have been extensively studied in secondary and late displacement. Our data are consistent with the literature regarding predictors for instability (LaMartina et al., 2015; Mackenney et al., 2006; Phillips and Al-Shawi, 2014; Wadsten et al., 2014; Walenkamp et al., 2015). For instance, LaMartina et al. (2015) found that dorsal comminution, age and anterior apposition were powerful predictors of the final radiographic outcome. A previous study from our institution found that anterior and dorsal comminution were significant predictors of early, secondary and late instability (Wadsten et al., 2014). Phillips and Al-Shawi (2014) also analysed early, secondary and late instability in a retrospective cohort and found that age and loss of anterior apposition were predictors of instability. Walenkamp et al. (2015) performed a meta-analysis of eight studies on secondary and late instability. They observed an increased risk of secondary and late displacement for patients aged >60–65 years, female sex and fractures with dorsal comminution. In a sub-analysis of late instability, Mackenney et al. (2006) found age and dorsal comminution as significant predictors.

Marginal secondary displacement in dorsal tilt and dorsal comminution were predictive factors for late deterioration in dorsal tilt. Other authors support our finding that fractures without dorsal support (i.e. dorsal comminution) will deteriorate in the direction of the comminution (LaMartina et al., 2015; Mackenney et al., 2006; Wadsten et al., 2014).

Several studies have reported that restoration of the congruence of the anterior cortex is vital to prevent late displacement (LaMartina et al., 2015; Phillips and Al-Shawi, 2014; Wadsten et al., 2017). Loss of anterior apposition was the only significant predictor for late deterioration in ulnar variance. Restoration of the anterior cortex prevents axial collapse, consistent with previous findings reported in the literature (Phillips and Al-Shawi, 2014). In our analysis, loss of anterior apposition and anterior comminution were strong predictors of late displacement.

The association between radial tilt and clinical outcome is weak (Mulders et al., 2018). Radial tilt was not a predictor of late displacement in our study. The most important instability features seem to be dorsal angulation and ulnar variance.

In conflict with previous studies, no significant association was seen between age or sex and late displacement (Hove et al., 1994; Walenkamp et al., 2015). One plausible explanation for this lack of association between age and late displacement is that we excluded patients greater than 75 years old.

DRF radiographic measurements are reliable and reproducible (Stirling et al., 2016). Johnson and Szabo (1993) found that the mean standard deviation for interobserver variability between surgeons was 3.2° for radial inclination on AP and 2.1° for dorsal tilt on lateral view (with the wrist elevated 15° from the table). In light of these findings, we defined marginal secondary displacement as a deterioration of 5° in concordance with Altissimi et al. (1994).

The study's major strengths are the prospective, multicentre study design, large sample size and routinely used measurements on standard radiographs that facilitate the applicability of the obtained results.

Inevitably, there are limitations to consider. First, radiograph rotation was not assessed, and the measurements were not adjusted to this factor. A change in rotation of 10° can sway the dorsal tilt by 2° (Johnson and Szabo, 1993). However, all images were performed according to a standardized protocol representing clinical routine in any orthopaedic department. One of the authors made all measurements with an intraclass correlation coefficient of good–excellent. Second, we did not record the number of patients excluded from the initial setting or who declined to participate in the study. We did not present clinical outcomes in the present cohort because this has been analysed and published previously (Wadsten et al., 2018).

In summary, marginal secondary displacement is a strong predictor of late displacement and malunion in DRFs. We suggest clinicians assess fracture alignment deterioration on radiographic follow-up and not outright accept alignment guidelines.

## Appendix 1

### Definitions

**Table:**

Early displacement	Displacement immediately after reduction or irreducible fracture.
Secondary displacement	Displacement occurs between the initial visit and 10–14 days after injury.
Late displacement	Displacement occurs between 10–14 days after injury and union.
Marginal secondary displacement	Deterioration (of ≥5° in dorsal tilt, ≥2 mm of ulnar variance or ≥5° of loss in radial inclination) between the initial visit and the 10–14 day follow-up, but with alignment still within an acceptable range.
Minimally displaced	Alignment within an acceptable range (anterior tilt <20°, dorsal tilt <10°, radial tilt >10° and positive ulnar variance <2 mm).
